# International partnerships in telehealth: healthcare, industry and education

**Published:** 2012-06-15

**Authors:** Frances Louise Finn, Conor O’Byrne

**Affiliations:** Waterford Institute of Technology, Ireland; Rigney Dolphin Group, Ireland

**Keywords:** telephone triage, hospital discharge programmes, partnerships

## Abstract

**Project background:**

In 2010 an internationally renowned American healthcare organisation partnered with Irish industry and higher education in Waterford with the goal to expand their telehealth services. Combining the skills and expertise of Nurse Consultants, Nurse Educators, IT Specialists and Healthcare Executives, these collaborative partnerships led to the delivery of telehealth services to North America from an Irish base, and to the development of new European telehealth programmes and telehealth training in Ireland. The telehealth service includes the provision of telephone triage, health information and advice, disease management and hospital discharge programmes to clients in Ireland, the UK and the USA. Telehealth nursing is an evolving specialty that requires the development of competence in key areas of information and communication technologies, assessment, triage and critical thinking in clinical decision-making within an environment where distance separates the nurse from the client.

**Aims and objectives:**

The aim of this paper is to report on the development, implementation and evaluation of the telehealth service with a focus on the telephone triage and advice service and the hospital discharge programmes. Objectives of this paper include describing this telehealth initiative with reference to the changing nature of global healthcare provision; discuss the educational strategy and accredited programme for training competent telehealth nurses; report the results of the evaluation of nurse performance in telephone triage and present the data relating to the impact of hospital discharge programmes on patient satisfaction and readmission rates.

**Methods and results:**

The evaluation of the telehealth training programme was undertaken six months post initial training and service commencement. One hundred triage and health information calls were reviewed, against best practice standards and programme learning outcomes, during a four-month period. Quantitative and qualitative data that demonstrates evidence of learning transfer from training to practice and the development of nurse competence from advanced beginner to levels of proficiency will be presented. The hospital discharge programmes have undergone continuous monitoring and reporting since commencement. This has enabled the collection of evidence that supports this brief telephone intervention as a method of reducing hopsital re-admissions and increasing patient satisfcation. Quantitative results will be presented and analysed in relation to the impact on patient satisfaction and readmission rates for patients discharged from cardiovascular, renal and digestive disease services.

**Conclusions:**

This project has demonstrated the effectiveness of partnerships in healthcare, industry and education in achieving the development, implementation and evaluation of international telehealth services. Initial education, training and ongoing support and development of nurses is essential for quality telehealth provision. Weekly call review, constructive feedback and reflection on practice are effective strategies for performance assurance and improvement. As a growing element of integrated healthcare, telehealth modules should be included in pre and post-registration nursing education curricula. Further collaboration between industry, healthcare and education are necessary in moving the telehealth agenda forward for the benefit of integrating services that impact positively on service users.

